# Screening for atrial fibrillation in care homes using pulse palpation and the AliveCor Kardia Mobile® device: a comparative cross-sectional pilot study

**DOI:** 10.1007/s11096-023-01672-z

**Published:** 2023-12-27

**Authors:** V. Savickas, A. J. Stewart, V. J. Short, A. Mathie, S. K. Bhamra, E. L. Veale, S. A. Corlett

**Affiliations:** 1 Medway School of Pharmacy, University of Kent and University of Greenwich, Chatham Maritime, UK; 2 Medway NHS Foundation Trust, Medway Maritime Hospital, Gillingham, UK; 3 Newton Place Surgery, Faversham, UK; 4 School of Allied Health Sciences, University of Suffolk, Ipswich, UK

**Keywords:** Atrial fibrillation, Care homes, Screening, Long-term care, Stroke

## Abstract

**Background:**

Atrial fibrillation (AF) is a major cause of stroke in older people. Exacerbated by age and co-morbidities, residents of care homes are more likely to develop AF and less likely to receive oral anticoagulants.

**Aim:**

To determine the prevalence of AF using the design and methodology of the Pharmacists Detecting Atrial Fibrillation (PDAF) study in a care home setting.

**Method:**

A cross-sectional AF screening pilot study within four UK care homes, three residential and one residential/nursing. Screening followed the original PDAF protocol: a manual pulse check, followed by a single-Lead ECG (_SL_ECG, AliveCor Kardia Mobile (KMD)) delivered by a pharmacist. All recorded _SL_ECG were reviewed by a cardiologist and any residents requiring follow-up investigations were referred to their general practitioner.

**Results:**

Fifty-three of 112 care home residents participated. From 52 _SL_ECGs recorded, the cardiologist interpreted 13.5% (7/52) as having possible AF of which 9.6% (5/52) were previously unknown. One resident with previously unknown AF received anticoagulation.

**Conclusion:**

This study has shown a need for AF screening in care homes and that elements of the PDAF screening protocol are transferable in this setting. Early diagnosis and treatment of AF are essential to reduce the risk of stroke in this population.

## Impact statements

- Whilst residents of care homes are more likely to be diagnosed with atrial fibrillation (AF), due to their older age, the feasibility of carrying out routine screening programmes is unknown.
- Care home residents are often underrepresented in research studies. Indeed, many barriers exist in conducting studies within this setting leading to a dearth of evidence to support healthcare interventions.
- This pilot study which used a previously tested AF screening protocol to determine the prevalence of undiagnosed AF in UK care homes found that residents were at much greater risk than the general population (9.6% vs. 1.3%).
- Whilst some practical challenges were observed in obtaining good quality traces these could be easily addressed with the adoption of more recently available technology. Pharmacists working within care homes could screen, diagnose, and optimise residents’ medicines to reduce the incidence and subsequent impact of AF related strokes in this vulnerable group.

## Introduction

The prevalence of atrial fibrillation (AF) and AF-related stroke is known to increase with age and disproportionately affects those over 80-years old (≥ 10% vs. 2.5% for AF and 24% vs. 1.8% for stroke) [1–4]. Older aged adults with multiple co-morbidities are concentrated in care homes. In a retrospective study of residential care homes in Wales, AF prevalence was 17.4% with an associated increased stroke risk (25.4% vs. 15.0%) [5]. Despite the benefits of oral anticoagulants (OACs) to reduce stroke risk, older people with increased frailty and polypharmacy are less likely to receive treatment [3, 6].

There is widespread debate regarding the benefits of screening for AF. Whilst routine screening is not recommended in the United Kingdom (UK) [7] opportunistic screening using pulse palpation or ECG rhythm strips is recommended for those ≥ 65 years in other countries [8, 9] and advocated for by the AF-SCREEN international collaboration [10].

The Pharmacists Detecting Atrial Fibrillation (PDAF) study demonstrated the clinical benefit of pharmacists undertaking opportunistic AF screening of ambulant patients aged ≥ 65 years in general practitioner (GP) practices [4]. There is a paucity of studies in care homes, a population at much higher risk of AF [11, 12]. The adoption of the PDAF screening programme in the care home setting may be viable, enabling residents to have access to timely diagnosis of AF and effective treatment.

### Aim

To determine the prevalence of AF using the design and methodology of the Pharmacists Detecting Atrial Fibrillation (PDAF) study [13] in a care home setting*.* A preliminary account of these findings has been reported previously [14].

### Ethics approval

Ethics approval was obtained from London Riverside Research Ethics Committee as an amendment to the PDAF study (17/LO/1650) on the 19^th^ September 2018.

## Method

### Study design and setting

This prospective cross-sectional study was conducted in four care homes affiliated to two GP practices: three residential and one mixed residential/nursing home within the UK between October 2018 and January 2019. Follow-up and baseline data was collected June 2019–June 2020. One clinical pharmacist, previously trained for the PDAF study [4, 13] to conduct AF screening, was accompanied by a GP who assessed each participant’s eligibility.

### Participant recruitment

Eligible participants were any residents of a participating care home, registered at one of the participating GP practices, aged ≥ 18 years and able to provide written informed consent. Exclusion criteria included having a pacemaker or defibrillator, lacking mental or physical capacity to provide informed consent, or receiving end-of-life care.

### Screening procedure

**Fig. 1:**
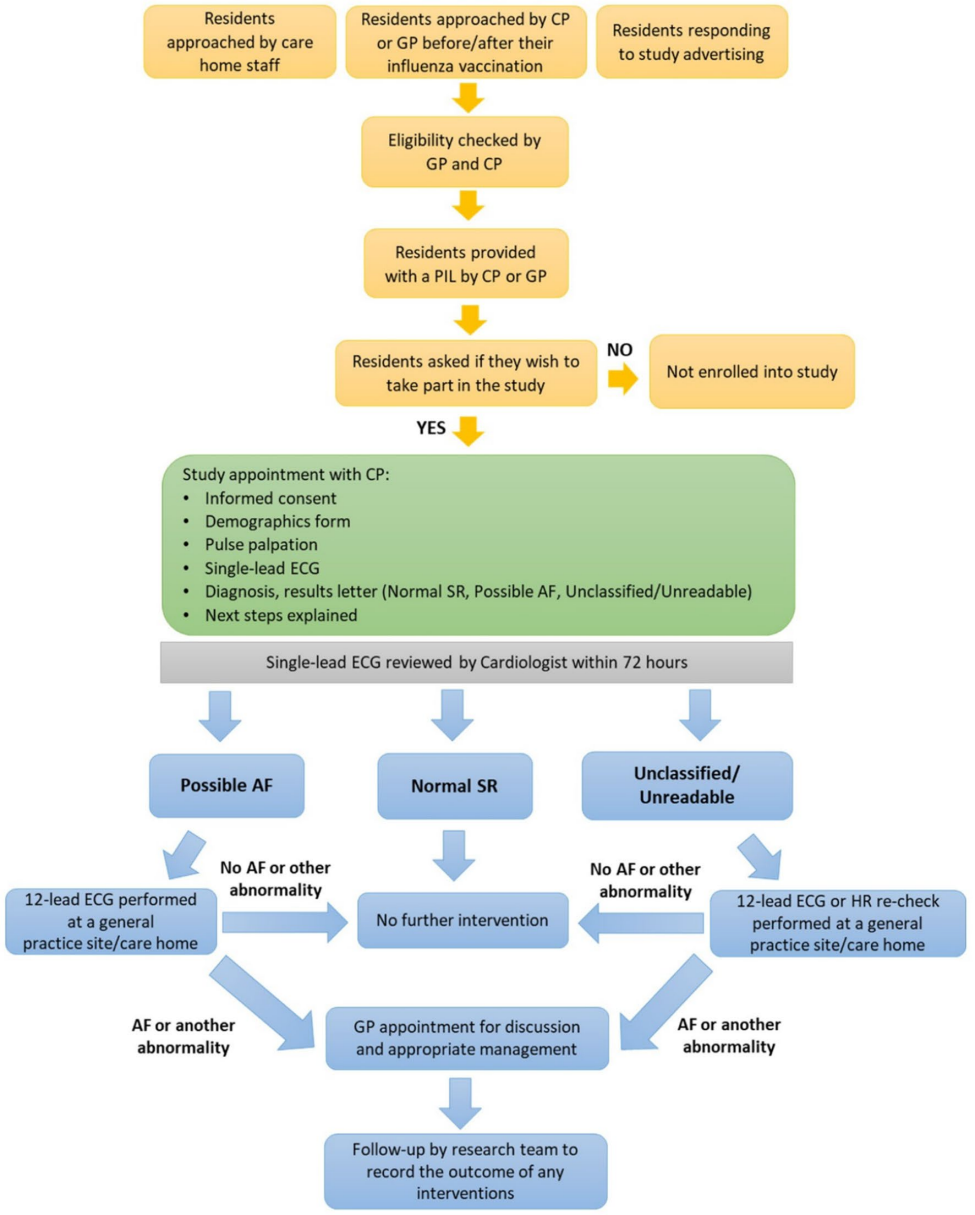
Care home participants recruitment, screening, and treatment flow chart. *AF: atrial fibrillation; CP: clinical pharmacist; ECG: electrocardiogram; GP: general practitioner; HR: heart rate; PIL: participant information leaflet; SR: sinus rhythm

Screening followed the original PDAF protocol [13], see Fig. 1. In brief, a manual radial/ulnar pulse palpation over 60 ﻿s was followed by a 30-s _SL_ECG using the AliveCor Kardia Mobile® device (KMD). The diagnosis following pulse palpation, and the KMD’s algorithm were noted. The cardiologist reviewed all _SL_ECGs within 72 h and those requiring further investigation were referred to their GP for a 12 Lead-ECG (_12L_ECG).

### Data analysis

All data were analysed using SPSS (V25). Categorical data were reported descriptively and continuous variables as median with inter-quartile range (IQR). The prevalence of AF, (known and unknown) expressed as mean (95% confidence intervals (CI)), was determined from the proportion of residents assigned ‘possible AF’ by the cardiologist’s review of the _SL_ECG. The yield of new AF was expressed as a percentage of participants with unknown AF who had the condition confirmed on _12L_ECG.

Sensitivity was determined from the ratio of true positives to the sum of the true positives and false negatives; specificity as the ratio of true negatives to the sum of true negatives and false positives; and accuracy from the ratio of the sum of true positives and true negatives to the total number of participants. The false discovery rate (FDR) was estimated from the ratio of false-positives to the sum of the true positives and false-positives.

## Results

### Participants

Residents assessed by the GP as eligible for the study (47.3%, 53/112) had a median age of 91 (interquartile range, IQR 86; 94) years. The majority were female (75.5%, 40/53) and all except one, who was South Asian, were of white British ethnicity. None smoked.

### Screening test results

**Fig. 2:**
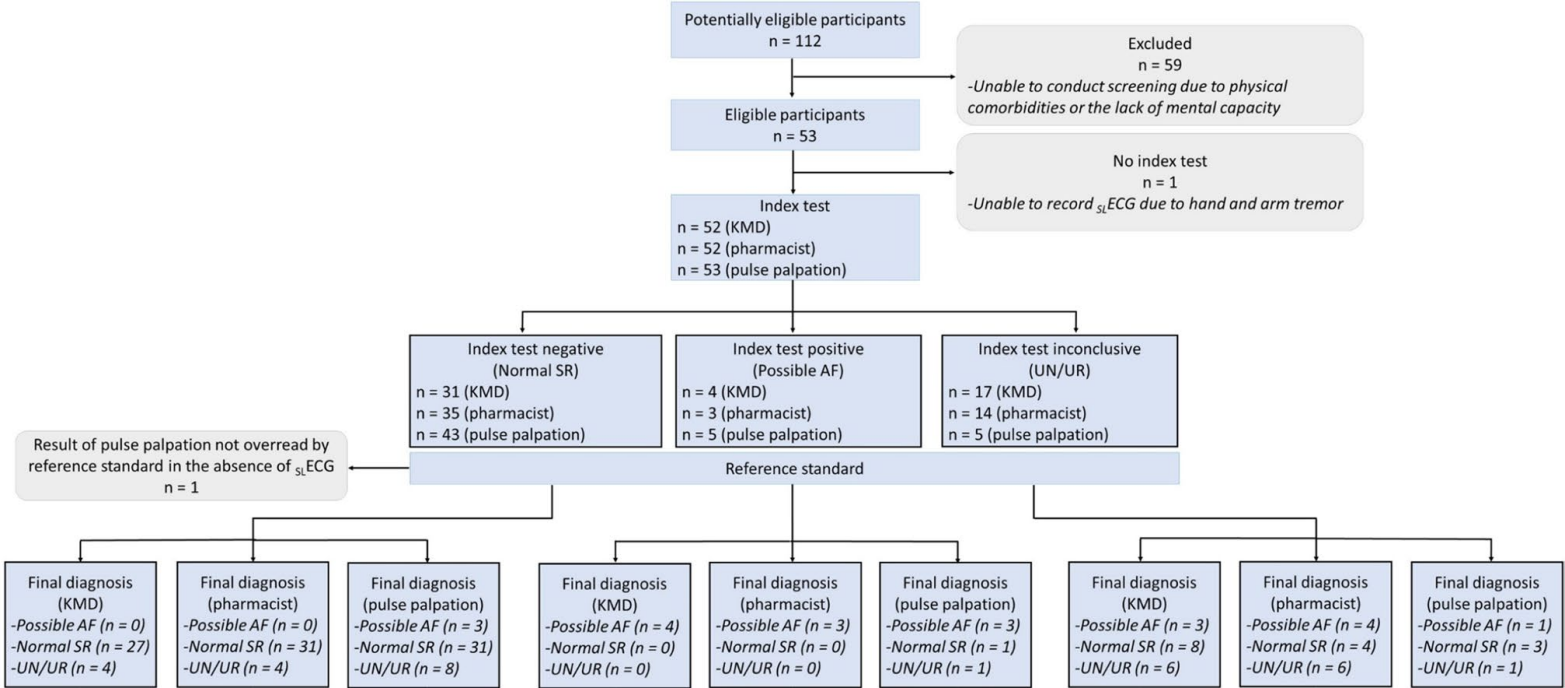
Summary of resident recruitment, screening and diagnostic classification by index test and reference standard (Cardiologist Interpretation of _SL_ECG). Adapted from Cohen et al. 2016. *AF: atrial fibrillation; *KMD*: Kardia Mobile Device; _*SL*_*ECG*: single-lead electrocardiogram; *SR*: sinus rhythm; *STARD*: standards for reporting diagnostic accuracy studies; *UN*: unclassified; *UR*: unreadable

The recruitment of participants and their diagnostic classification by each index test is summarised in Fig. 2. A _SL_ECG was obtained for 52 participants, 1/53 could not be recorded due to severe hand tremor. Participants’ median heart rate was 76 (68; 82; n = 52). _SL_ECG quality was categorised as poor in 27% of cases, and acceptable or excellent in the remainder. The cardiologists interpreted 35 (67.3%) _SL_ECGs as being ‘normal sinus-rhythm’ (SR), seven (13.5%) as ‘possible AF’, and the remainder as ‘unclassified’, comprising inconsistent or unidentifiable p waves (9.6%, 5/52), possible bundle branch block (BBB) (7.7%, 4/52) and possible atrial flutter (1.9%, 1/52). The total prevalence of AF was 13.5% (95% CI, 5.6–25.8); 9.6% (5/52) as previously ‘unknown’ AF.

Residents with ‘possible AF’ had a median BMI of 23.8 (19.9; 26.9) kg/m^2^ and CHA_2_DS_2_VASc and HAS-BLED scores of 3 (3; 6) and 1 (1; 2). Reported comorbidities were hypertension (5/7, 71.4%), renal disease (3/7, 42.9%), diabetes mellitus (2/7, 28.6%), peripheral vascular disease (2/7, 28.6%), thyroid disease (1/7, 14.3%) and transient ischaemic attack (1/7, 14.3%). Three (42.9%) were male (Table 1).

**Table 1:** Demographic of cardiologist-confirmed ‘Possible AF’ cases in care homes

Baseline demographics	Participants with possible AF in care homes (n = 7)	All eligible participants in care homes (n = 53)
Age, years	90 [87; 94]	91 [86; 94]
Male	3 (42.9)	13 (24.5)
Current alcohol drinker	0 (0.0) (n = 1)	10 (47.6) (n = 21)
Alcohol, units/week	0 (0.0) (n = 1)*	1.0 [1.0; 6.5] (n = 9)
Current smoker	0 (0.0)	0 (0.0) (n = 50)
Height, cm	177.8 [161.1; 180.0]	162.0 [154.3; 170.2] (n = 40)
Weight, kg	69.0 [61.4; 80.0]	64.2 [60.0; 78.1] (n = 43)
BMI, kg/m^2^	23.8 [19.9; 26.9]	25.1 [20.9; 29.2] (n = 38)

Enhanced demographics	Participants with possible AF in care homes (n = 7)	
CHA_2_DS_2_VASc score	3.0 [3.0; 6.0]	
HAS-BLED score	1.0 [1.0; 2.0]	
Hypertension	5 (71.4)	
Renal disease	3 (42.9)	
Diabetes mellitus	2 (28.6)	
Thyroid disease	1 (14.3)	
Transient ischaemic attack	1 (14.3)	
Ischaemic heart disease	0 (0.0)	
Heart failure	0 (0.0)	
Intracranial bleed	0 (0.0)	
Peripheral vascular disease	2 (28.6)	
COPD	0 (0.0)	

Continuous variables are expressed as a median [interquartile range]. Categorical variables are expressed as the number of participants (% total of the group). *AF atrial fibrillation, *COPD* chronic obstructive pulmonary disease, *BMI* body mass index, *GP* general practitioner

The KMD algorithm produced no false-positives, with the three additional ‘possible AF’ cases identified by the cardiologist assigned as ‘unclassified’. However, eight participants (15.4%) labelled as ‘unclassified’ by the algorithm were assigned ‘normal SR’ by the cardiologist leading to possible unnecessary referrals for _12L_ECG. Pulse palpation produced two false-positives, one confirmed as ‘normal SR’ and one as ‘unclassified’, and four false-negative cases, three assigned as normal SR and one as ‘unclassified’.

The overall diagnostic accuracy of pulse palpation was lower although no significant difference was observed (*p* > 0.05).

### Follow-up outcomes

Of the 15 care home residents who were referred for a follow-up _12L_ECG, 66.7% (10/15) received this procedure within a median time of 24.5 (15.5; 50.8) days. One resident did not require a _12L_ECG due to a known BBB, one participant died and three did not respond. For those who received a _12L_ECG, one (1.9%, 1/52) remained in AF and was prescribed a direct-acting OAC. The remaining residents were either in SR (9.6%, 5/52), had BBB (5.8%, 3/52) or atrioventricular block (1.9%, 1/52).

## Discussion

### Statement of key findings

The prevalence of AF was five-fold higher than that observed in the general population [1], with almost 10% of participating residents suspected to have previously undiagnosed AF. Despite the KMD algorithm having a lower diagnostic sensitivity than previously observed in the PDAF study [4] this test was most closely aligned with the cardiologist’s diagnostic classification, demonstrating superiority over pulse palpation. The quality of the _SL_ECG was rated poor and difficult to interpret in almost one third of traces, due to baseline artefacts caused by age-related tremor, resulting in some unnecessary _12L_ECG referrals. Residents diagnosed by the cardiologist as ‘possible AF’ or ‘unclassified’ by the cardiologist were offered a _12L_ECG with a median follow-up of 25 days; substantially longer than the 16 days noted in the PDAF study [4]. Only one participant with previously unknown AF received OAC therapy.

### Strengths and weaknesses

Our study is the first in the UK to explore the feasibility of pharmacists conducting single-time point, opportunistic AF screening for an at-risk population based in a care home using a digital _SL_ECG device identifying residents at risk of ischaemic stroke, who would benefit from OAC therapy.

A significant limitation of the study, however, is the small sample size, with over half of the residents not eligible to participate due to pronounced physical or mental incapacity. In addition, participants were recruited from four care homes within one geographical area, which together limits the generalisability of these findings in ascertaining prevalence and baseline demographic for this population. Stakeholder feedback to inform feasibility for the intervention is missing.

### Interpretation

This study has provided evidence, utilising an established AF screening protocol [13] which aligns closely with the AF-SCREEN guidelines [10], of the high prevalence of undiagnosed AF in care-home residents (9.6% versus 1.3% in GP practices) at increased risk of ischaemic stroke. The extended delay in receiving the _12L_ECG following screening and receiving appropriate treatment such as OAC therapy, highlights the evidenced barriers associated with care home residents accessing routine external healthcare [15]. Insight into the practical challenges of conducting AF screening in care homes residents, such as cognitive and physical impairments, has been demonstrated.

### Further research

This study excluded over half of all residents due to severe physical and/or cognitive impairments, despite AF being linked to cognitive decline and dementia [16]. A wider study to measure AF prevalence incorporating not only this previously excluded at-risk group but wider ethnic minority groups, utilising alternative screening options, such as modified blood pressure monitors [17, 18] or the 6-Lead KMD (AliveCor. Inc) to circumvent the issue of poor-quality traces, and generalisability of the data, is required. In addition, future studies should explore the acceptability of identifying and initiating appropriate AF treatment in care homes from multiple stakeholders. Clinicians are less likely to offer OAC therapy despite obvious health benefits, due to the perceived risks associated with residents having multiple comorbidities, a short life expectancy and an elevated risk of bleeding [19, 20]. A much larger and adequately powered randomised trial of this AF screening programme would provide the evidence needed for adoption in UK care homes.

## Conclusion

Residents of care homes are at increased risk of developing AF and suffering from an AF-related stroke. The true benefit of any AF screening programme cannot be realised unless residents are followed up efficiently and considered for stroke prevention with an OAC on a case-by-case basis. Service provision in care homes can be extremely variable, despite the implementation of the ‘Enhanced Health in Care Homes’ framework [15, 21]. Pharmacists are experts in medicine optimisation and increasingly are qualifying as prescribers [22]. A single timepoint pharmacist-led AF screening and anticoagulation clinic in care homes could be one pathway for the early diagnosis and treatment of AF in a rapidly growing, at-risk and often overlooked, population [23].
